# Exosomal miRNAs in autoimmune skin diseases

**DOI:** 10.3389/fimmu.2023.1307455

**Published:** 2023-12-01

**Authors:** Ri Zhang, Yujia Wei, Tingmei Wang, Xiaoqi Nie, Zeqi Shi, Yunhua Deng, Dong Li

**Affiliations:** Department of Dermatology, Tongji Hospital, Tongji Medical College, Huazhong University of Science and Technology, Wuhan, China

**Keywords:** exosome, miRNA, autoimmune skin disease, immunology, skin

## Abstract

Exosomes, bilaterally phospholipid-coated small vesicles, are produced and released by nearly all cells, which comprise diverse biological macromolecules, including proteins, DNA, RNA, and others, that participate in the regulation of their biological functions. An increasing number of studies have revealed that the contents of exosomes, particularly microRNA(miRNA), play a significant role in the pathogenesis of various diseases, including autoimmune skin diseases. MiRNA is a class of single-stranded non-coding RNA molecules that possess approximately 22 nucleotides in length with the capability of binding to the untranslated as well as coding regions of target mRNA to regulate gene expression precisely at the post-transcriptional level. Various exosomal miRNAs have been found to be significantly expressed in some autoimmune skin diseases and involved in the pathogenesis of conditions via regulating the secretion of crucial pathogenic cytokines and the direction of immune cell differentiation. Thus, exosomal miRNAs might be promising biomarkers for monitoring disease progression, relapse and reflection to treatment based on their functions and changes. This review summarized the current studies on exosomal miRNAs in several common autoimmune skin diseases, aiming to dissect the underlying mechanism from a new perspective, seek novel biomarkers for disease monitoring and lay the foundation for developing innovative target therapy in the future.

## 1 Introduction

MicroRNA (miRNA), first discovered within nematodes by Ambro et al. (1) in 1993, is a class of single-stranded non-coding RNA molecules typically consisting of approximately 22 nucleotides in length. MiRNA binds to the 3’ untranslated region (UTR) and coding region of target mRNA through base pairing, leading to either degradation or inhibition of translation of the target mRNA (2), enabling precise control of gene expression at the post-transcriptional level (3). MiRNA is involved in various cellular activities, including proliferation, differentiation, apoptosis, and metabolism (4). It has emerged as a potential biomarker and therapeutic target for numerous diseases, making it a prominent focus of research in the medical field in recent years (5).

Exosomes, vesicular structures with a diameter ranging from 40 to 160nm enclosed within a bilayer phospholipid membrane, contain diverse biological molecules, including nucleic acids, lipids and proteins. They are widely distributed and can be generated by almost all cells and detected in various body fluids (6–9).

Unlike freely circulating miRNA in body fluids, miRNA within exosomes resists RNA degradation by ribonucleases, making it more stable (10). Furthermore, exosomal miRNA exhibits potential homing properties (11) and selective enrichment, enabling disease occurrence, progression and relapse monitoring (12), which is critical for clinical management of autoimmune diseases.

Autoimmune skin diseases are defined as autoimmune diseases characterized by excessive activation of the immune system leading to abnormal immune reactions and responses against self-antigens that are normally tolerated, including but not limited to skin involvement, such as vitiligo, psoriasis, systemic lupus erythematosus and dermatomyositis. In recent years, there has been a steady increase in the incidence and prevalence of autoimmune skin diseases, accounting for an essential part of the global disease mortality and economic burden. The uncertainty of etiology, the complexity of pathogenesis, and the unpredictable nature of disease progression have posed significant challenges to patients treating and managing autoimmune skin diseases, significantly impacting patients’ quality of life. Therefore, searching for new therapeutic targets and identifying biomarkers that can predict disease progression and treatment effectiveness is crucial. As mentioned above, exosomal miRNA can be a promising choice for its function and nature. A comprehensive map illustrating how exosomal miRNAs are involved in autoimmune skin diseases by regulating transcripts, pathways, immune system differentiation, and their interactions with terminal cells such as keratinocytes, fibroblasts, and immune cells is no doubt important yet still lacking. Here, we reviewed recent evidence on the role of exosomal miRNAs within autoimmune skin diseases and discussed their impact on these diseases (**Table 1**), aiming to facilitate a better understanding of the pathogenetic mechanisms of autoimmune skin diseases and clinical management.

**Table 1:** Summary of exosomal miRNAs profiles of indicated autoimmune skin diseases.

Disease	Exo-miRNA	State in disease group/specific cells	Origin of exosome	Target cell	Target gene	Significance	References
vitiligo	miR-21-5p	up	plasma	melanocyte	*STAB1*	inhibit melanogenesis	(13)
	miR-487b-3p	down	plasma	-	*-*	biomarker for monitoring disease progression	(14)
	miR-200C	down	primary keratinocyte	melanocyte	*SOX1*	promote melanogenesis, potentially mediated by the *Wnt* pathway	(15)
	miR-493-3p	up	plasma	keratinocyte	*hnRNPU*	miR-493-3p/*hnRNPU*/ *COMT*/*DA* axis is involved in the initial damage of melanocytes	(16)
psoriasis	miR-381-3p	up	keratinocyte	CD4-positive T-cell	*UBR5* and *FOXO1*	induce Th1 and Th17 polarization and promote psoriasis development	(17)
	246 miRNAs	up/down	plasma	-	-	provide abundant circulating exosomal miRNAs, target genes and signaling pathways for further research	(18)
	let-7b-5p and miR-30e-5p	down	plasma	-	-	biomarkers for arthritis in psoriasis patients	(19)
	miR-151a-3p, miR-199a-5p, miR-370-3p, miR-589-5p, and miR-769-5p	up	plasma	-	-	participate in the common pathogenesis of psoriasis vulgaris, psoriatic arthritis, rheumatoid arthritis and gouty arthritis	(20)
atopic dermatitis	miR-147	down	plasma	HaCaT cell	*TLSP*	exert protective effects by inhibiting *TLSP* expression	(21)
	25 miRNAs	up/down	plasma	-	-	biomarkers for psychological stress	(22)
severe drug eruption	miR-375-3p	up	plasma	keratinocyte	*XIAP*	promote apoptosis in keratinocytes by downregulating *XIAP*	(23)
	miR-18a	up	plasma	keratinocyte	*BCL2L10*	induce keratinocyte apoptosis by downregulating *BCL2L10*	(24)
	miR-4488	up	peripheral blood mononuclear cell	HaCaT cell	-	promote HaCaT cell apoptosis	(25)
	miR-96-5p	down					
dermatomyositis	miR-125a-3p, miR-1246, and miR-3614-5p	up	plasma	human skeletal muscle myocyte		clinical biomarkers and therapeutic targets	(26)
	miR-4488	up/down	plasma	-	*DDX39B*	biomarkers for DM-ILD-*MDA5* Ab (+)	(27)
	miR-1228-5p				*ZBTB22* and *MDM 2*		
	10 miRNAs	up/down	neutrophil	-	-	modulate *PI3K-Akt*, *MAPK*, *AMPK*, and *FoxO* pathways	(28)
	10 miRNAs	up/down	plasma	human aortic endothelial cell	59 genes	involved in vessel-related inflammation in juvenile dermatomyositis	(29)
scleroderma	17 miRNAs	up/down	plasma	normal human dermal fibroblast	-	identify 17 fibrosis-related miRNAs in systemic sclerosis	(30)
	22 miRNAs	up/down	neutrophil	human dermal fibroblast and human dermal microvascular endothelial cell	-	uncover the role of neutrophil in systemic sclerosis	(31)
	miR-214	down	bone marrow mesenchymal stem cell	fibroblast	*IL-33*	inhibit *IL-33*/*ST2* signaling	(32)
	miR-196b-5p	up	mesenchymal stem cell	fibroblast	*COL1A2*	alleviate skin fibrosis by inhibiting *COL1A2* expression	(33)
	miR-151-5p	up	mesenchymal stem cell	bone marrow mesenchymal stem cell	*IL-4Ra*	improve bone loss by targeting *IL-4Ra*	(34)
systemic lupus erythematosus	miR-574	up	plasma	plasmacytoid dendritic cell	*IFN-a*	structures resembling *IFN*-inducing motifs promote pDCs maturation and secretion	(35)
	miR-155	up	peripheral blood B-cell	B-cell	*SHIP-1*	regulate cell proliferation and activation of peripheral blood B-cells through targeting SHIP-1	(36)
	miR-19b	down	peripheral blood mononuclear cell	CD4-positive T-cell	*KLF13*	result in the differentiation of CD4-positive T-cells into Tregs and reduce the production of inflammatory cytokines	(37)
	miR-146a	down	plasma	bone marrow mesenchymal stem cell	*TRAF6*	rescue the senescence of BM-MSCs via inhibiting TRAF6/NF-κB pathway	(38)
	miR-146-5p	up	umbilical cord blood mesenchymal stem cell	macrophage	*NOTCH1*	suppress excessive inflammatory responses and protect against alveolar injury by inhibiting *NOTCH1* expression	(39)
	miR-195-5p	down	urine	-	*CXCL10*	biomarker for lupus nephritis	(40)

## 2 Exosomal miRNAs in several autoimmune skin diseases

### 2.1 Exosomal miRNAs in vitiligo

Vitiligo is an acquired pigmentary skin disorder that involves the participation of various innate and adaptive immune cells, leading to damage to melanocytes in the skin and hair follicles (41), resulting in depigmented patches.

Several researches have yielded positive results that circulating exosomal miRNAs contribute to vitiligo’s pathogenesis. Zhang et al. (13) co-cultured circulating exosomes from vitiligo patients with the human melanocyte cell line PIG1. They observed the inhibition of melanogenesis, decreased tyrosinase activity, and altered expression of genes related to melanogenesis in melanocytes. Furthermore, they detected significantly higher expression of miR-21-5p in exosomes from vitiligo patients and confirmed that miR-21-5p inhibits melanogenesis, as evidenced by changes in the levels of tyrosinase and tyrosinase-related protein 1 (*TYP1*) and tyrosinase-related protein 2 (*TYP2*). Luo et al. (14) found that circulating exosomal miR-487b-3p in advanced-stage vitiligo patients was significantly downregulated before glucocorticoid treatment but recovered to normal levels after intervention. Enrichment analysis suggested that this miRNA primarily affects metabolic pathways.

Additionally, skin keratinocytes (42) and fibroblasts (43) are involved in this disease’s abnormal immune environment that promotes local T-cell infiltration by secreting *CXCL9, CXCL10*, and various cytokines. Moreover, melanocytes appear to be not solely victims of the abnormal immune response but also participants in initiating the immune dysfunction (44). Zhao et al. (15) found that melanocytes exhibited a significant decrease in melanin content and tyrosinase activity after being cultured with exosomes from vitiligo patients’ skin lesional keratinocytes. Furthermore, they discovered that miR-200C, downregulated in these exosomes, could promote melanogenesis, potentially mediated by the inhibition of *SOX1* to activate the *Wnt* pathway. Li et al. (16) performed high-throughput sequencing and correlation analysis of circulating exosomal miRNAs from segmental vitiligo patients and healthy controls with disease progression and staging, screened and expanded the specimens to verify that miR-493-3p, which was highly expressed in circulating exosomes of the patients as well as keratinocytes of the lesions. They subsequently demonstrated *in vitro* the miR-493-3p-*hnRNPU-COMT-DA* axis on the initial damage of melanocytes. However, there are no current studies on exosomal miRNAs referring to the role of fibroblasts or melanocytes initiating abnormal immunity, which might be worth investigating in the future to uncover the mechanism of vitiligo from multidimensional perspectives.

### 2.2 Exosomal miRNAs in psoriasis

Psoriasis is a chronic inflammatory skin disease characterized by abnormal activation and infiltration of T-cells and excessive proliferation of keratinocytes, clinically manifesting red plaques and papules covered with thick silver-white scales. Th17 cells and *IL-17A*/*IL-23* play a crucial role in the immune dysfunction in psoriasis (45). Jiang et al. (17) discovered that exosomes derived from keratinocytes treated with psoriasis cytokines (*IL-17A*, *IL-22*, *IFN-γ*, *TNF-α*) can induce the differentiation of CD4-positive T-cells into Th1 and Th17 cells and upregulate the expression of various cytokines including *IL-17A*, *IL-17F*, *IL-22*, *IL-23*, *IL-36*, *IFN-γ*, and *TNF-α*. MiR-381-3p was found to upregulate the expression of the indicated cytokines in CD4-positive T-cells. *UBR5* and *FOXO1* were identified as critical downstream target genes, playing essential roles in the immune response in psoriasis.

Chen et al. (18) sequenced circulating exosomal miRNAs in 15 patients with psoriasis vulgaris and 15 healthy controls and identified 246 differentially expressed miRNAs. Subsequently, they discovered that enrichment analysis could enrich some target genes in inflammatory metabolic pathways. This study facilitated other researchers to select circulating exosomal miRNAs and target genes of interest at the subsequent step of investigating in psoriasis.

In addition to skin involvement, joints can also be affected, known as psoriatic arthritis (PsA). Pasquali et al. (19) identified 15 circulating exosomal miRNAs from 14 patients with psoriasis vulgaris (PsV) and 15 PsA patients. After expanding the sample size for validation along with regression analysis, they found that the expression levels of let-7b-5p and miR-30e-5p were negatively correlated with PsA group, which might be possible biomarkers for PsA. Chen et al. (20) extracted plasma exosomes from 15 PsV patients, 30 PsA patients, 15 patients with rheumatoid arthritis, 15 patients with gouty arthritis, and 15 healthy controls and identified five miRNAs (hsa-miR-151a-3p, hsa-miR-199a-5p, hsa-miR-370-3p, hsa-miR-589-5p, and hsa-miR-769-5p) that mainly participate in the common pathogenesis of these four diseases by affecting inflammation and bone metabolism.

### 2.3 Exosomal miRNAs in atopic dermatitis

Atopic dermatitis (AD) is a chronic inflammatory skin disease characterized by intense itching, alternating acute episodes, and remissions, significantly affecting patients’ quality of life. Crosstalk between keratinocytes and various immune cells results in skin barrier impairment via secreting diversified cytokines, such as *TNF-α* and *IFN-γ* (46). Shi et al. (21) detected significantly downregulated levels of miR-147 in the plasma, lesional tissues of a mouse model of AD and HaCaT cell models compared to the negative control group. The expression of miR-147 was negatively correlated with *TLSP* and *VEGFA*, two vascular growth-related factors considered important in the pathogenesis of AD (47). Treatment with *TNF-α*/*IFN-γ* decreased the viability of HaCaT cells, upregulated *TLSP* and *VEGFA* expression, and promoted cell apoptosis. However, overexpression of miR-147 reversed these damaging processes in HaCaT cells. Furthermore, they found that exosomes from adipose-derived stem cells overexpressing miR-147 exerted similar protective effects and inhibited *TLSP* expression (21). These results indicate exosomal miR-147 as a potential target for AD therapy.

Besides, some studies have indicated a correlation between the onset of atopic dermatitis and psychological stress. Moreover, it has been demonstrated that psychotherapy can alleviate some patients’ symptoms (48). Sung et al. (22) performed sequencing and differential analysis of plasma-derived neuronal exosomes from AD mouse models, identifying 9 significantly upregulated and 16 considerably downregulated miRNAs, which can be utilized to unveil mechanism of AD regarding psychological factors.

### 2.4 Exosomes miRNAs in severe drug eruption

Drug eruption, one of the most common adverse drug reactions, manifests as inflammatory skin and mucosal lesions with possible systemic involvement. Stevens-Johnson Syndrome (SJS), Toxic Epidermal Necrolysis (TEN), Acute Generalized Exanthemata’s Pustulosis (AGEP) and Drug Reaction with Eosinophilia and Systemic Symptoms (DRESS) are collectively known as severe drug eruptions characterized by large-scale death of keratinocytes (49). High mortality rates of severe drug eruptions urge dermatologists to excavate disease mechanisms and develop improved therapies. exosomes miRNA helps better understand the pathogenesis of severe drug eruptions that remain unclear.

Zhang et al. (23) identified upregulation of miR-375-3p in exosomes from patients with SJS and TEN. Overexpression of miR-375-3p in primary human keratinocytes reduced cell viability and promoted apoptosis via downregulating *XIAP*. Furthermore, they observed a positive correlation between the expression levels of miR-375-3p and the affected body surface area and epidermal necrosis score in patients with severe drug eruptions. Salinas et al. (24) investigated circulating exosomal miRNAs of patients with severe drug eruptions (9 cases of DRESS, 8 cases of SJS/TEN) and identified 24 significantly upregulated miRNAs, which were predominantly involved in T-cell activation, cell apoptosis, and inflammation processes. From these findings, they verified differential overexpression of miR-18a, consistent with previous research (50) that showed significant upregulation of miR-18a in the skin lesions and plasma of TEN patients, and it was found to induce keratinocyte apoptosis by downregulating *BCL2L10*. Suthumchai et al. (25) observed cytotoxic effects on HaCaT cells by exosomes secreted by peripheral blood mononuclear cells from 12 patients with SJS/TEN and identified upregulated miR-4488 and downregulated miR-486-5p, miR-96-5p, and miR-132-3p. Furthermore, both overexpression of miR-4488 and reduced expression of miR-96-5p promoted HaCaT cells apoptosis.

### 2.5 Exosomal miRNAs in dermatomyositis

Dermatomyositis (DM) is an autoimmune disease characterized by edematous purplish-red patches on the upper eyelids, as well as flat brownish-red papules on exposed areas.

Weakness and myalgia are the main manifestations of muscle involvement. Li et al. (26) confirmed the upregulation of has-miR-125a-3p, has-miR-1246, and has-miR-3614-5p in circulating exosomes from untreated DM patients and in human skeletal muscle myoblasts cells stimulated by these exosomes, which returned to normal levels after antirheumatic treatment. Furthermore, these three miRNAs were related to the cellular autophagy pathway and positively correlated with specific indicators in DM patients, such as serum creatine kinase levels and myositis antibody titers, suggesting them as potential biomarkers for DM.

The existence of anti-MDA5 antibodies was related to interstitial lung disease and a worse prognosis in DM patients (51). Zhong et al. (27) sequenced circulating exosomal miRNAs from 5 patients with DM accompanied by interstitial lung disease and positive anti-*MDA5* antibody, 5 patients with DM without myositis antibodies, and 5 healthy controls and found significant differences in has-miR-4488 and hsa-miR-1228-5p in all three comparisons. *H2AFX* and *MDM2* were identified as two essential hub genes that may be involved in the pathogenesis of DM. Nonetheless, this study didn’t focus on the mechanism underlying the distinction between MDA5 positive and MDA5 negative DM patients.

Neutrophil/lymphocyte ratio in peripheral blood was found associated with disease activity, lung involvement, and overall survival in DM patients (52). Additionally, proteases within neutrophil cytoplasm seem to participate in muscle inflammation (53). Li et al. (28) validated 10 differentially expressed miRNAs in neutrophils-derived exosomes from DM patients’ peripheral blood and determined *PI3K*-*Akt*, *MAPK*, *AMPK*, and *FoxO* as the main downstream signaling pathways, demonstrated and partially explained the role of neutrophil in DM.

In addition, evidences indicate a close relationship between the pathogenesis of DM and vascular changes (54). Jiang et al. (29) identified 10 differentially expressed circulating exosomal miRNAs in adolescent DM patients and 59 differentially expressed genes after co-culturing exosomes with human aortic endothelial cells. Through reciprocal prediction, they found specific downregulated genes in the patient group corresponding to upregulated miRNAs in the exosomal sequencing data, providing some miRNA-target gene axes for future research.

### 2.6 Exosomal miRNAs in scleroderma

Scleroderma, a chronic autoimmune skin disease characterized by abnormal activation of fibroblasts leading to progressive skin and visceral fibrosis, can be classified into two types: localized cutaneous scleroderma and systemic scleroderma, also known as systemic sclerosis (SSc) (55).

Wermuth et al. (30) validated the upregulation of 9 pro-fibrotic miRNAs and the downregulation of 8 anti-fibrotic miRNAs in exosomes derived from plasma of SSc patients and detected an upregulation of type I collagen fibers and fibronectin in normal human dermal fibroblasts stimulated by these exosomes, indicating the pro-fibrotic function of circulating exosomal miRNAs in SSc.

It has been found that neutrophil-derived exosomes from the peripheral blood of patients can inhibit endothelial cell proliferation and migration (56). Li et al. (31) discovered 22 differentially expressed miRNAs in neutrophil-derived exosomes from the peripheral blood of SSc patients and identified the involvement of the *Wnt*, *IL-23*, *NOTCH* and *AMPK* pathways. Further co-culturing the exosomes above with primary human dermal fibroblasts and human dermal microvascular endothelial cells from healthy individuals validated the presence of negative correlations between specific miRNAs and target genes associated with these molecular pathways, suggesting neutrophil as one of culprits in SSc.

Mesenchymal stem cells (MSCs) are a type of pluripotent stem cell derived from various tissues such as bone marrow, adipose tissue, placenta, and umbilical cord (57). These cells have demonstrated beneficial effects in improving the manifestations of scleroderma through their anti-inflammatory and anti-fibrotic properties (58), and it is believed that these effects are primarily mediated through the production of extracellular vesicles including exosomes (59). Xie et al. (32) identified downregulation of miR-214 in the peripheral blood of the SSc patients and discovered that bone marrow- MSC-derived exosomes could transport miR-214 to fibroblasts and inhibit their proliferation, migration, and expression of fibrosis-related genes via inhibiting *IL-33*/*ST2* signaling. Baral et al. (33) found that injection of exosomes derived from MSCs could alleviate skin fibrosis in bleomycin-induced SSc mice. They further identified upregulated miR-196b-5p and overexpression of this miRNA in mouse fibroblasts downregulated *COL1A2* expression, speculating that miR-196b-5p may play a role in the anti-fibrotic effects of MSC-derived exosomes. Chen et al. (34) discovered that MSCs transplantation could improve bone loss in systemic sclerosis mice by modulating the differentiation of recipient bone marrow-MSCs probably attributed to miR-151-5p derived from MSC-derived exosomes by targeting IL-4Ra, consistent with a previous study identifying IL-4 as a suspicious pathway (60).

These findings exhibited therapeutic potential of MSC-derived exosomal miRNAs for SSc.

### 2.7 Exosomal miRNAs in systemic lupus erythematosus

Systemic lupus erythematosus (SLE) is a complex autoimmune skin disease involving multiple systems. The characteristic skin lesions of SLE include edematous butterfly rash on the face, discoid rash, vasculitis-like lesions in the distal limbs, oral ulcers, and easily breakable hair at the frontal hairline.

Type I interferon plays a crucial role in the pathogenesis of SLE (61). Salvi et al. (35) demonstrated that circulating exosomal miRNAs with structures resembling interferon-inducing motifs from SLE patients can promote the maturation of human peripheral blood plasmacytoid dendritic cells(pDCs) and their secretion of type I interferon and other pro-inflammatory factors.

In addition to SSc, MSCs were also found therapeutic in SLE by modulating adaptive immunity. Patients exist various autoantibodies in their bodies, exemplified by anti-double-stranded DNA and anti-Sm antibodies (62), demonstrating the dominance of humoral immunity in SLE. Zhao et al. (36) found significantly higher numbers of peripheral blood B-cells in untreated SLE patients compared to healthy individuals, validated the upregulation of miR-155 and confirmed the binding relation between miR-155 and *SHIP-1*. Additionally, exosomes derived from umbilical cord blood-derived MSCs could upregulate *SHIP-1* expression in B-cells and inhibit cell proliferation, activation, and promotion of apoptosis.

However, cellular immunity shouldn’t be easily neglected in SLE. Tu et al. (37) determined that miR-19b was downregulated while *KLF13* was upregulated in peripheral blood mononuclear cells of patients with SLE and verified the binding and negative correlation between miR-19b and *KLF13*. They further discovered that umbilical cord blood-derived MSCs could enrich miR-19b in exosomes, resulting in the differentiation of CD4-positive T-cells into Tregs and reducing the production of inflammatory cytokines.

On the contrary, MSCs might be victims surrounded by abnormal immune microenvironment in SLE. Dong et al. (38) discovered that plasma and plasma exosomes derived from SLE patients could promote the senescence of bone marrow MSCs, accelerate the degradation of *IκBα*, phosphorylation and translocation of *p65*, and exosomal miR-146a was downregulated. Based on previous studies and prediction websites, miR-146a is predicted to target and inhibit the expression of *TRAF6*, indicating that plasma-derived exosomal miR-146a may regulate the senescence of bone marrow MSCs through negative regulation of the *TRAF6*/*NF-κB* pathway.

Studies of exosomal miRNAs also focus on lupus nephritis and lung damage in SLE. Chen et al. (39) found that exosomes derived from umbilical cord blood-derived MSCs could alleviate diffuse alveolar hemorrhage in SLE mice and demonstrated that exosomes from umbilical cord blood-derived MSCs could transport miR-146-5p to target and inhibit the expression of *NOTCH1*, thus promoting the polarization of M2 macrophages, leading to the suppression of excessive inflammatory responses and protection against alveolar injury. Cheng et al. (40) demonstrated downregulation of miR-195-5p in the urine of lupus nephritis patients and its negative modulation of *CXCL10*, consistent with their initial result based on online available data. Furthermore, this miRNA was negatively correlated with urinary protein, renal damage, serum complement levels, and disease severity.

## 3 Discussion

Autoimmune skin diseases impose significant burdens on patients, families, and society. Despite the availability of various treatment options, the lack of a clear understanding of the underlying mechanisms of the diseases, coupled with their tendency for progression and relapse, often leads to unsatisfactory outcomes in terms of treatment and management. Exosomes, extracellular vesicles that transport important molecules such as miRNA to recipient cells, play a crucial role in modulating gene expression and altering cellular functions. Studies of exosomal miRNA can provide insights into the intrinsic mechanisms of diseases, facilitate the identification of potential biomarkers and molecular targets, and lay the foundation for disease monitoring and the development of novel therapies and drugs from a new perspective. However, there is limited foundational research on exosomal miRNA in autoimmune skin diseases, with a scarcity of clinical studies and low levels of evidence. Therefore, more standardized research designs and larger-scale studies to explore the role of exosomal miRNA in autoimmune skin diseases are warranted in the future.

## Author contributions

RZ: Writing - original draft, Data curation. YW: Writing - original draft, Writing - review & editing. TW: Writing - review & editing. XN: Writing - review & editing. ZS: Writing - review & editing. YD: Writing - review & editing. DL: Conceptualization, Writing - review & editing.
